# Glyceraldehyde-derived pyridinium (GLAP) evokes oxidative stress and inflammatory and thrombogenic reactions in endothelial cells via the interaction with RAGE

**DOI:** 10.1186/s12933-014-0162-3

**Published:** 2015-01-08

**Authors:** Takanori Matsui, Eriko Oda, Yuichiro Higashimoto, Sho-ichi Yamagishi

**Affiliations:** Department of Pathophysiology and Therapeutics of Diabetic Vascular Complications, 67 Asahi-machi, Kurume, 830-0011 Japan; Department of Chemistry, Kurume University School of Medicine, Kurume, 830-0011 Japan

**Keywords:** AGEs, RAGE, GLAP, Aptamer, Oxidative stress

## Abstract

**Background:**

We have previously shown that serum levels of glyceraldehyde-derived advanced glycation end products (Gly-AGEs) are elevated under oxidative stress and/or diabetic conditions and associated with insulin resistance, endothelial dysfunction and vascular inflammation in humans. Further, Gly-AGEs not only evoke oxidative and inflammatory reactions in endothelial cells (ECs) through the interaction with a receptor for AGEs (RAGE), but also mimic vasopermeability effects of AGE-rich serum purified from diabetic patients on hemodialysis. These observations suggest that Gly-AGE-RAGE system might be a therapeutic target for vascular complications in diabetes. However, since incubation of glyceraldehyde with proteins will generate a large number of structurally distinct AGEs, it remains unclear what type of AGE structures could mediate the deleterious effects of Gly-AGEs on ECs.

**Aims and Methods:**

Therefore, in this study, we examined (1) whether glyceraldehyde-derived pyridinium (GLAP), one of the Gly-AGEs generated by the incubation of lysine with glyceraldehyde, elicited reactive oxygen species (ROS) generation and inflammatory and thrombogenic gene expression in human umbilical vein ECs (HUVECs) via the interaction with RAGE and (2) if DNA aptamers raised against Gly-AGEs or GLAP (AGE-aptamer or GLAP-aptamer) inhibited the binding of GLAP to RAGE and subsequently suppressed the harmful effects of GLAP on HUVECs.

**Results:**

GLAP stimulated ROS generation in a bell-shaped manner; GLAP at 10 μg/ml increased ROS generation in HUVECs by 40%, which was blocked by the treatment with RAGE-antibody (RAGE-Ab). Ten μg/ml GLAP significantly up-regulated mRNA levels of RAGE, monocyte chemoattractant protein-1, intercellular adhesion molecule-1, vascular cell adhesion molecule-1 and plasminogen activator inhibitor-1 in HUVECs, which were also suppressed by RAGE-Ab. AGE-aptamer or GLAP-aptamer significantly blocked these deleterious effects of GLAP on HUVECs. Moreover, quartz crystal microbalance analyses revealed that GLAP actually bound to RAGE and that AGE-aptamer or GLAP-aptamer inhibited the binding of GLAP to RAGE.

**Conclusions:**

The present study suggests that GLAP might be a main glyceraldehyde-related AGE structure in Gly-AGEs that bound to RAGE and subsequently elicited ROS generation and inflammatory and thrombogenic reactions in HUVECs. Blockade of the GLAP-RAGE interaction by AGE-aptamer or GLAP-aptamer might be a novel therapeutic strategy for preventing vascular injury in diabetes.

## Background

Advanced glycation end products (AGEs) are formed by the Maillard process, a non-enzymatic reaction between reducing sugars and amino groups of proteins, lipids or nucleic acids, that contributes to the aging of proteins [1-3]. This process begins with the conversion of reversible Schiff base adducts to more stable Amadori products [1-3]. Over the course of days to weeks, the Amadori products undergo further rearrangement reactions such as dehydration and condensation to form irreversibly cross-linked macroprotein derivatives termed AGEs [1-3]. The formation and accumulation of AGEs in various tissues have been known to progress at a physiological aging and at an accelerated rate under hyperglycemic and/or oxidative stress conditions such as diabetes [1-3]. There is accumulating evidence that AGEs and the receptor for AGEs (RAGE) interactions play a central role in the development and progression of vascular complications [4-8]. Among various reducing sugar-modified AGEs, we have previously shown that serum levels of glyceraldehyde-derived advanced glycation end products (Gly-AGEs) are elevated under oxidative stress, inflammatory and/or diabetic conditions and associated with insulin resistance, endothelial dysfunction, vascular inflammation and decreased number and migratory activity of endothelial progenitor cells in humans [9-14]. Further, Gly-AGEs not only evoke oxidative and inflammatory reactions in endothelial cells (ECs) through the interaction with RAGE, but also mimic vascular permeability effects of AGE-rich serum purified from diabetic patients on hemodialysis [15-20]. These observations suggest that Gly-AGE-RAGE system might be a therapeutic target for preventing vascular complications in diabetes. However, since incubation of glyceraldehyde with proteins will generate a large number of structurally distinct AGEs, it remains unclear what type of AGE structures could mediate the deleterious effects of Gly-AGEs on ECs.

In 1990s, an *in vitro*-selection process called systematic evolution of ligands by exponential enrichment (SELEX) was developed to screen aptamers, single-stranded DNA or RNA molecules that can bind with high affinity and specificity to a wide range of target proteins [21]. Due to its small size, non-immunogenicity and ease of modification compared to conventional monoclonal antibodies, a couple of aptamers have been used in the clinical fields as a tool for neutralizing function of various proteins [22,23]. Indeed, we have recently found that high-affinity DNA aptamer directed against Gly-AGEs (AGE-aptamer) inhibits the binding of Gly-AGEs to RAGE and subsequently protects against the Gly-AGE-induced inflammatory reactions in ECs and mesangial cells, and suppresses the progression of nephropathy in an animal model of type 2 diabetes, neointimal hyperplasia in balloon-injured rat carotid arteries and melanoma growth in nude mice [24-27]. These findings indicate that AGE-aptamer could be a valuable tool for blocking the deleterious effects of Gly-AGEs in both cell culture and animal models. In this study, to identify a main AGE structure in Gly-AGEs that could mediate the biological actions, we examined (1) whether glyceraldehyde-derived pyridinium (GLAP), one of the Gly-AGEs generated by the incubation of lysine with glyceraldehyde, elicited reactive oxygen species (ROS) generation and inflammatory and thrombogenic gene expression in human umbilical vein ECs (HUVECs) via the interaction with RAGE and (2) if AGE-aptamer or DNA aptamer directed against GLAP (GLAP-aptamer) inhibited the binding of GLAP to RAGE and resultantly suppressed the harmful effects of GLAP on HUVECs.

## Methods

### Materials

D, L-Glyceraldehyde, bovine serum albumin (BSA) (essentially fatty acid free and essentially globulin free, lyophilized powder), and *N*-acetyl-L-lysine were purchased from Sigma (St. Louis, MO, USA). Carboxy-H_2_DFFDA from Life Technologies Japan (Tokyo, Japan).

### Cells

HUVECs were cultured in endothelial basal medium supplemented with 2% fetal bovine serum, human fibroblast growth factor β, heparin, human epidermal growth factor and hydrocortisone according to the supplier’s instructions (Lonza Japan Ltd., Tokyo, Japan). GLAP or carboxy-H_2_DFFDA treatment was carried out in a medium lacking epidermal growth factor and hydrocortisone.

### Synthesis and purification of GLAP

GLAP was synthesized according to a slightly modified method of Usui et al. [28]. In brief, glyceraldehyde (0.2 M) and *N*-acetyl-L-lysine (0.1 M) were dissolved in 0.2 M sodium phosphate buffer (pH 7.4), and incubated at 37°C for a week. The reaction mixture was filtered with a polyvinylidene difluoride membrane filter (0.22 mm, Millipore, Bedford, MA, USA), and then put on a C8 column on preparative reversed phase high-performance liquid chromatography (HPLC). HPLC was done with a quaternary gradient pump PU-2089 plus (JASCO Co. Ltd., Tokyo, Japan) and monitoring at 215 nm with the UV–VIS spectrophotometric detector UV-2075 plus (JASCO Co., Ltd. Tokyo, Japan) under the following conditions: Column: COSMOSIL 5C8-AR-300 column (250 × 20 mm I.D., Nacalai Tesque Inc., Kyoto, Japan). Elution: isocratic of 25 mM sodium phosphate buffer (pH 7.0) from 0 to 30 min and a linear gradient of 0-40% acetonitrile containing 25 mM sodium phosphate buffer (pH 7.0) from 30 to 60 min. Flow rate: 2 ml/min. Temperature: ambient.

### Preparation of antibody directed against human RAGE (RAGE-Ab)

RAGE-Ab, which recognizes the amino acid residues 167–180 of human RAGE protein, was used for neutralizing assays and prepared as described previously [29].

### Immobilizing GLAP on agarose beads

GLAP was covalently coupled via carboxy groups to amine groups on CarboxyLink Coupling Gel (Pierce, Rockford, IL, USA) with crosslinker 1-Ethyl-3-(3-dimethylaminopropyl) carbodiimide according to the manufacturer's instructions.

### SELEX

Preparation and selection of GLAP-aptamer were performed as described previously [30]. Synthetic DNA templates (106-mer) with 56 random nucleotides, 5’-AGCTCAGAATGGATCCAAACGCTCA-(N)56-TTCGACATGAGAATTCGGCCGGATC-3’, were amplified over 12 cycles of polymerase chain reaction (PCR) (94°C, 20 seconds, 52°C, 20 seconds, and 72°C, 20 seconds) using 5’- and 3’-primers. From the purified PCR products, single-stranded DNAs were obtained by an additional 45 cycles of PCR using 5’-primer only. The single-stranded DNA pool was then loaded onto a GLAP-immobilized agarose column (3 mm × 10 mm). After 30 min, bound DNA was eluted with 0.3 mL of phosphate-buffered saline (PBS) at 95°C. To remove single-stranded DNAs that had non-specifically bound to agarose beads, the eluted DNA was passed through a control agarose column without GLAP immobilization. The recovered single-stranded DNA was amplified by PCR and used as the input DNA for the next selection. After repeating the SELEX procedure seven times, sequences of cloned single-stranded DNAs were determined using an automatic sequencer (ABI PRISM; Perkin-Elmer Applied Biosystems, Foster, CA, USA). DNA aptamers are susceptible to degradation by nucleases [25]. This will limit their applications for real samples, such as blood and tissues. To solve this issue, we modified aptamers with phosphorothioate linkage at each three bases.

### Preparation of Gly-AGEs

Gly-AGEs were prepared as described previously [15]. In brief, BSA was incubated under sterile conditions with glyceraldehyde for 7 days. Then, unbounded sugars were removed by dialysis against PBS. Preparations were tested for endotoxin using Endospecy ES-20S system (Seikagaku Co., Tokyo, Japan); no endotoxin was detectable.

### Preparation and selection of AGE-aptamer

Preparation and selection of AGE-aptamer were performed as described previously [24]. Sequences of AGE-aptamer and control DNA aptamer (Control-aptamer) are below. AGE-aptamer; 5’-TGTAGCCCGAGTATCATTCTCCATCGCCCCCAGATACAAG-3’, Control-aptamer; 5’-TTCGGCCTGGGGGCGGCCAGTTCGGGTCCAGTCGCGGGAG-3’. AGE- and Control-aptamer were modified with phosphorothioate as described previously [24].

### Measurement of intracellular ROS generation

Intracellular formation of ROS was detected using a fluorescent probe carboxy-H_2_DFFDA (Life Technologies Japan., Tokyo, Japan) as described previously [31]. In brief, HUVECs were incubated with 0.1% dimethyl sulfoxide (DMSO) in the presence or absence of 10 μM carboxy-H_2_DFFDA for 1 hr. Then the cells were washed with PBS three times, and treated with or without the indicated concentrations of GLAP in the presence or absence of 5 μg/ml RAGE-Ab, 100 nM AGE-aptamer, Control-aptamer or GLAP-aptamer. After 10 min, intracellular superoxide generation was measured with an ARVO X3 fluorescent plate reader (PerkinElmer Japan, Yokohama, Japan). ROS production was calculated by subtracting the fluorescence for cells pre-incubated with DMSO only from that with carboxy-H_2_DFFDA.

### Real-time reverse transcription-PCR (RT-PCR)

HUVECs were treated with or without the indicated concentrations of GLAP in the presence or absence of 5 μg/ml RAGE-Ab, 100 nM AGE-aptamer, Control-aptamer or GLAP-aptamer for 4 hr. Then total RNA was extracted with NucleoSpin RNA kit (Takara Bio　Inc., Shiga, Japan) according to the manufacturer’s instructions. Quantitative real-time RT-PCR was performed using Assay-on-Demand and TaqMan 5 fluorogenic nuclease chemistry (Life Technologies Japan Ltd., Tokyo, Japan) according to the supplier’s recommendation. IDs of primers for human RAGE, monocyte chemoattractant protein-1 (MCP-1), intercellular adhesion molecule-1 (ICAM-1), vascular cell adhesion molecule-1 (VCAM-1), plasminogen activator inhibitor-1 (PAI-1), and 18S gene were Hs00542592_g1, Hs00234140_m1, Hs99999152_m1, Hs01003372_m1, Hs01126604_m1, and Hs03003631_g1, respectively.

### Secondary structure analyses of GLAP- and AGE-aptamers

Secondary structure analyses of GLAP- and AGE-aptamers were performed using the computer program mFold supported by Zuker [32].

### Binding affinities of GLAP to RAGE, AGE-aptamer or GLAP-aptamer

The binding affinities of GLAP to extracellular AGE-binding v-domain of RAGE (vRAGE), AGE-aptamer, GLAP-aptamer or Control-aptamer were measured using sensitive 27-MHz quartz crystal microbalance (QCM) (Affinix Q; Initium Inc., Tokyo, Japan) under the presence or absence of 2% fetal bovine serum (Lonza Group Ltd., Basel, Switzerland) as described previously [24,25]. In brief, recombinant vRAGE, AGE-aptamer or GLAP was immobilized on a QCM surface. After adding GLAP or GLAP-aptamer to reaction vessel, the time course of the frequency decrease of bound vRAGE, bound AGE-aptamer or bound GLAP on the QCM was monitored. The binding affinities of GLAP to vRAGE, AGE-aptamer or GLAP-aptamer were calculated from curve fitting to the QCM frequency decrease as described previously [24,25].

### Statistical analysis

All values were presented as mean ± standard error. Student’s *t*-test was performed for statistical comparisons; p < 0.05 was considered significant.

## Results

**Figure 1:**
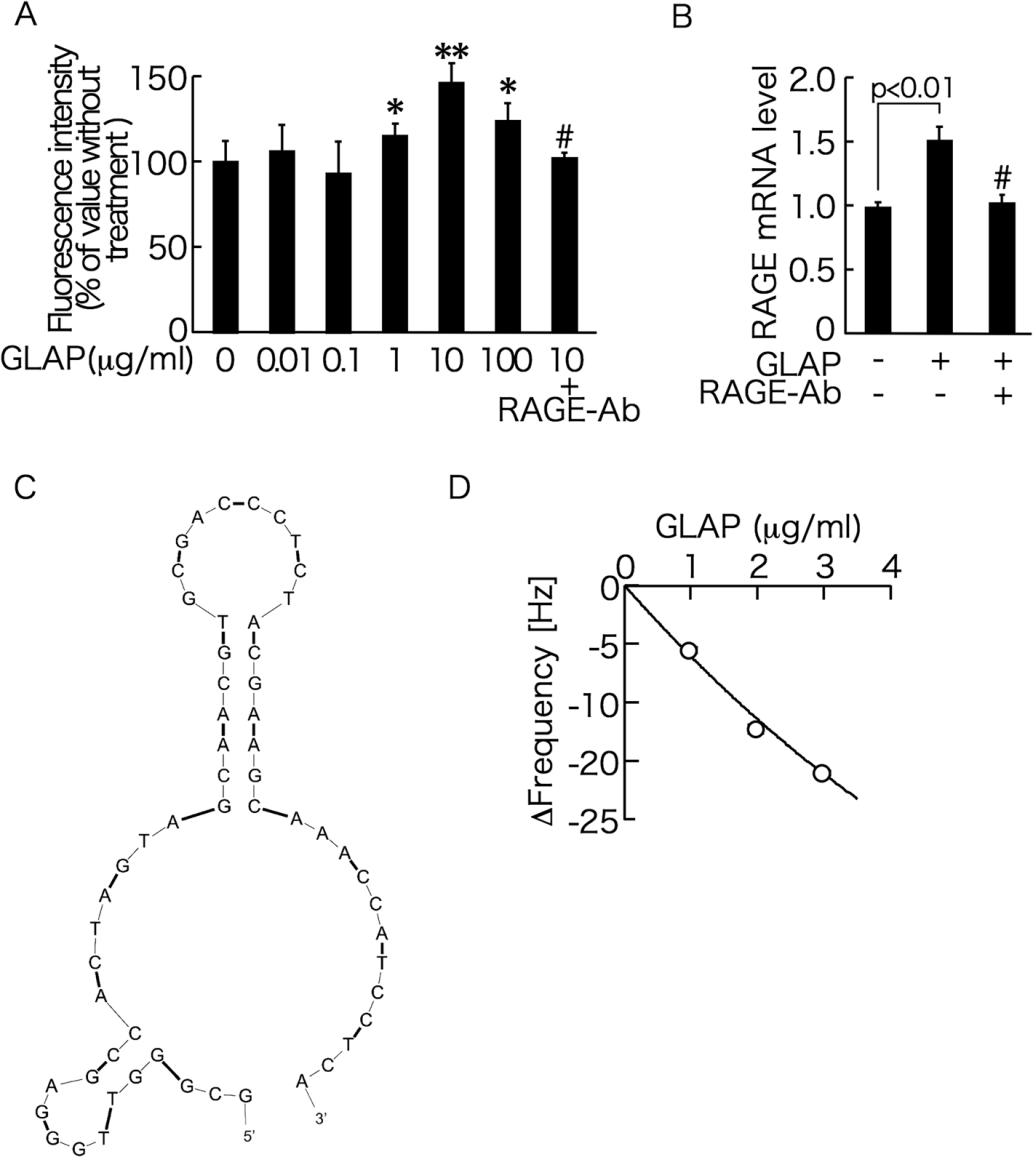
**Effects of GLAP on ROS generation (A) and RAGE gene expression in HUVECs (B), secondary structure of GLAP-aptamer (C), and binding affinity of GLAP to immobilized vRAGE (D). (A)** HUVECs were incubated with 0.1% DMSO in the presence or absence of 10 μM carboxy-H_2_DFFDA for 1 hr, and then treated with or without the indicated concentrations of GLAP in the presence or absence of 5 μg/ml RAGE-Ab. After 10 min, intracellular superoxide generation was measured. * and **, p < 0.05 and p < 0.01 compared to the value without treatment, respectively. #, p < 0.05 compared to the value with 10 μg/ml GLAP. *N* = 4 per group. **(B)** HUVECs were treated with or without 10 μg/ml GLAP in the presence or absence of 5 μg/ml RAGE-Ab for 4 hr. Then total RNAs were extracted, transcribed and amplified by real-time PCR. Data were normalized by the intensity of 18S mRNA-derived signals and then related to the value without treatment. #, p < 0.05 compared to the value with 10 μg/ml GLAP. *N* = 3-6 per group. **(C)** Secondary structure of GLAP-aptamer. Phoshorothioate linkages are shown as bold line. **(D)** GLAP at 1, 2 and 3 μg/ml was added on vRAGE-immobilized reaction vessel. The time course of the frequency decrease of bound vRAGE on the QCM was monitored. Two-independent experiments were performed.

We first examined the effects of GLAP on ROS generation in HUVECs. As shown in Figure 1A, GLAP increased superoxide generation in HUVECs in a bell-shaped manner; ROS generation began to be induced by 1 μg/ml GLAP and reached a maximum by 10 μg/ml GLAP, and the peak value was about 1.4-fold higher than the basal level. RAGE-Ab completely blocked the increase in ROS generation evoked by 10 μg/ml GLAP. Since ROS generation was maximumly induced by 10 μg/ml GLAP, we chose the concentration of GLAP at 10 μg/ml for the following experiments.

We next investigated the effects of GLAP on RAGE gene expression in HUVECs. As shown in Figure 1B, GLAP up-regulated RAGE mRNA levels in HUVECs, which was also completely blocked by the treatment with RAGE-Ab.

Secondary structure of GLAP-aptamer with phosphorothioate linkage was provided in Figure 1C. Phoshorothioate linkages are shown as bold line. These aptamers showed typical stem-loop or bulge-loop structure with cytosine-rich sequences such as ACC(C) or (C)CCA.

QCM is a technique to detect a mass change on an electrode at nanogram level from the resonance frequency change; when molecules bound on oscillating quartz crystal, oscillating frequency decreases in proportional to binding amount of molecules on the surface [33]. As shown in Figure 1D, GLAP bound to vRAGE in a dose-dependent manner; dissociation constant (*K*_D_) value was 5.9 × 10^−5^ M.

**Figure 2:**
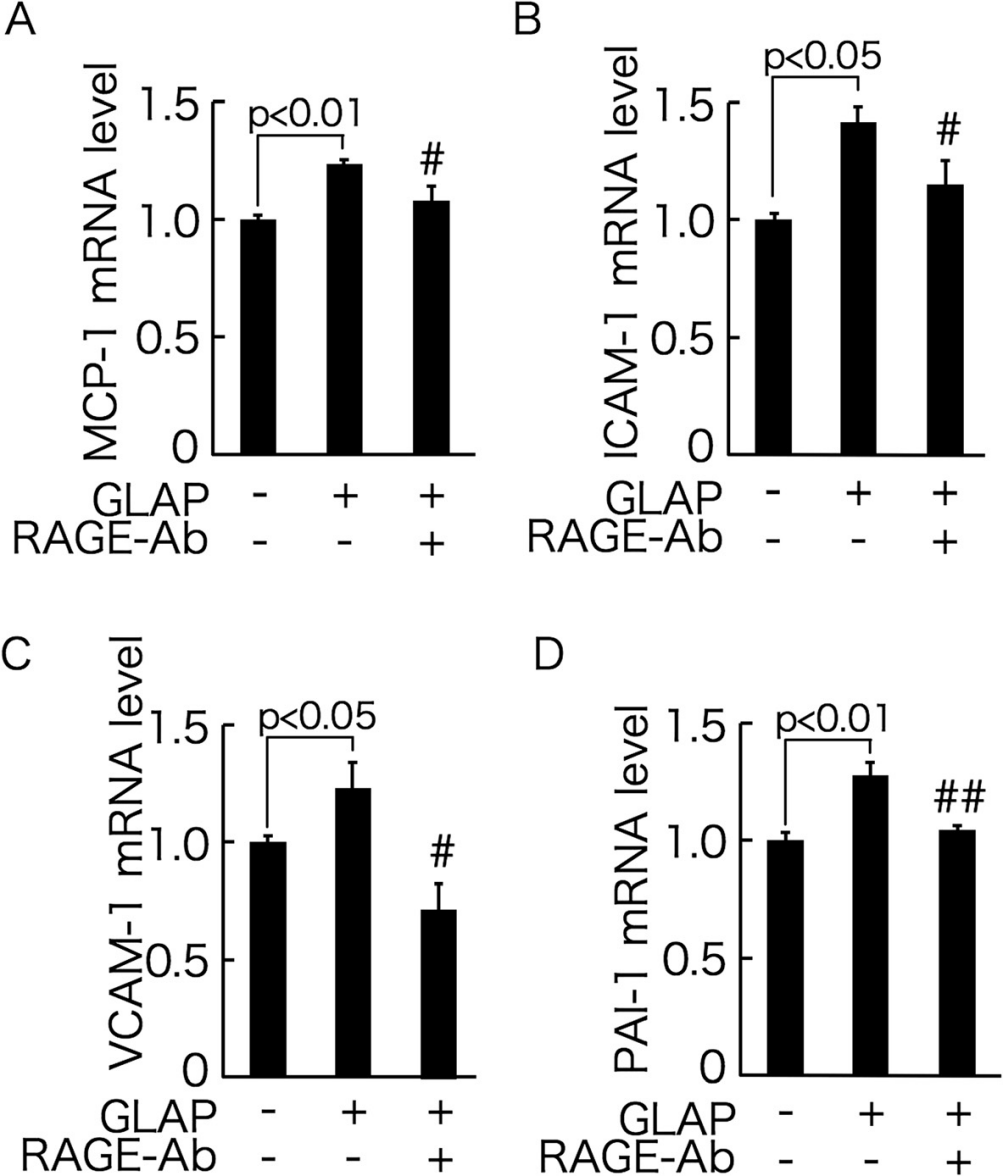
**Effects of GLAP on MCP-1 (A), ICAM-1 (B), VCAM-1 (C), and PAI-1 gene expression in HUVECs (D).** HUVECs were treated with or without 10 μg/ml GLAP in the presence or absence of 5 μg/ml RAGE-Ab for 4 hr. Then total RNAs were extracted, transcribed and amplified by real-time PCR. Data were normalized by the intensity of 18S mRNA-derived signals and then related to the value obtained with control. # and ##, p < 0.05 and p < 0.01 compared to the value with 10 μg/ml GLAP alone, respectively. *N* = 3-6 per group.

We further examined whether GLAP elicited inflammatory and thrombogenic gene expression in HUVECs. As shown in Figure 2A-D, GLAP significantly up-regulated MCP-1, ICAM-1, VCAM-1 and PAI-1 mRNA levels in HUVECs, which was suppressed by the treatment with RAGE-Ab.

**Figure 3:**
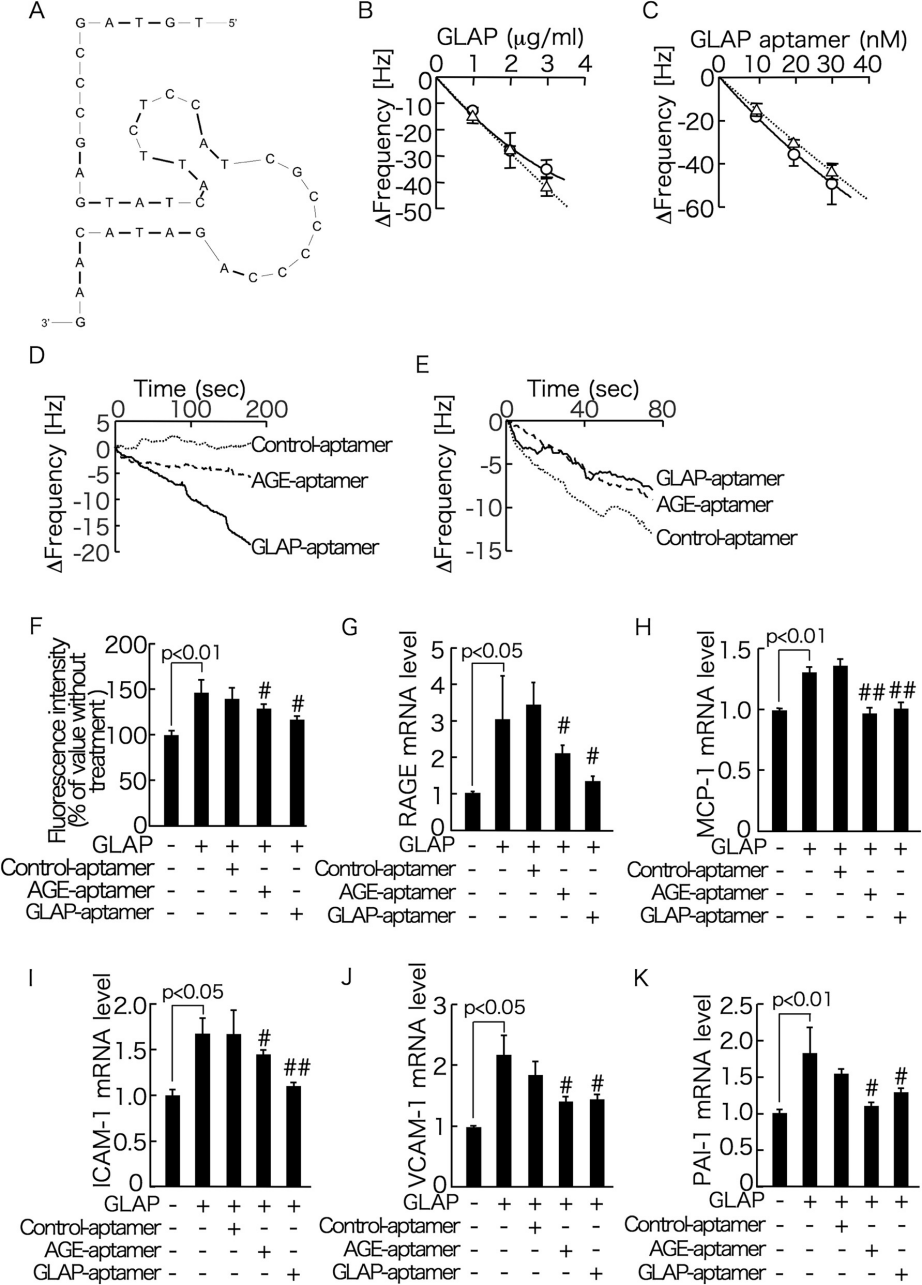
**Secondary structure of AGE-aptamer (A), binding affinities of GLAP to AGE-aptamer, GLAP-aptamer, or vRAGE (B-E) and effects of AGE- or GLAP-aptamer on inflammatory and thrombogenic gene expression in GLAP-exposed HUVECs (F-K). (A)** Secondary structure of AGE-aptamer. Phoshorothioate linkages are shown as bold line. AGE-aptamer **(B)**, GLAP (**(C)** and **(D)**) or recombinant vRAGE **(E)** was immobilized on a QCM surface. After adding GLAP (**(B)** and **(E)**), GLAP-aptamer **(C)** or each aptamer **(D)** to reaction vessel, the time course of the frequency decrease of bound AGE-aptamer **(B)**, bound GLAP (**(C)** and **(D)**), or bound vRAGE **(E** on the QCM was monitored. Under no serum conditions, solid line and circule. Under 2% fetal bovine serum conditions, dashed line and triangle. *N* = 3 per group. (F)-(K) HUVECs were treated with or without 10 μg/ml GLAP in the presence or absence of 100 nM each aptamer for 4 hr. Then total RNAs were extracted, transcribed and amplified by real-time PCR. Data were normalized by the intensity of 18S mRNA-derived signals and then related to the value obtained with control. # and ##, p < 0.05 and p < 0.01 compared to the value with 10 μg/ml GLAP alone, respectively. *N* = 4 per group.

DNA aptamers specific for GLAP were isolated by an *in vitro*-selection process, SELEX, from a pool of ~10^15^ different nucleic acid sequences. In this study, 34 clones were sequenced from the pool of selected single-stranded DNAs. The length of sequence randomized in the original library was 56 nucleotides; however, the length of the inserts of the clones obtained ranged from 55 to 57 nucleotides, probably due to insertion or deletion during PCR amplification. Sequence of GLAP-aptamer used for the present experiments was GCGGGTTGGGAGCCACTAGTAGCAACGTGCGACCCTCTACGAAGCAAACCATCCTCA. Secondary structure of AGE-aptamer with phosphorothioate linkage was provided in Figure 3A. Phoshorothioate linkages are shown as bold line.

As shown in Figure 3B-E, AGE-aptamer or GLAP-aptamer, but not Control-aptamer, bound to GLAP with *K*_D_ values of 3.3 × 10^−5^ M and 0.26 × 10^−5^ M, respectively, and inhibited the binding of GLAP to vRAGE. The bindings of these aptamers to GLAP were not affected under 2% fetal bovine serum conditions that mimic the state in functional experiments of aptamers. Moreover, GLAP-induced increase in ROS generation as well as RAGE, MCP-1, ICAM-1, VCAM-1 and PAI-1 mRNA levels of HUVECs were significantly inhibited by the treatment with AGE-aptamer or GLAP-aptamer, but not Control-aptamer (Figure 3F-K).

## Discussion

### GLAP-induced EC damage

GLAP, 3-hydroxy-5-hydroxymethyl-pyridinium adduct, is a major compound found in Gly-AGEs, and its plasma levels as well as the contents in tail collagen or brain of diabetic rats are extremely increased compared with normal rats [28,34,35]. However, its pathophysiological role in vascular damage in diabetes is largely known. In other words, it remains unclear whether GLAP could mainly mediate the deleterious effects of Gly-AGEs on HUVECs. Therefore, we first examined whether GLAP mimicked the effects of Gly-AGEs in HUVECs. In this study, we found that (1) GLAP increased ROS generation in HUVECs in a bell-shaped manner, (2) GLAP up-regulated RAGE mRNA levels in HUVECs, (3) GLAP actually bound to vRAGE, and (4) RAGE-Ab completely prevented the GLAP-induced ROS generation and RAGE gene induction in HUVECs. These findings suggest the pathological crosstalk between ROS generation and RAGE gene induction in GLAP-exposed HUVECs. So, engagement of RAGE with GLAP could induce ROS generation, which led to up-regulate RAGE expression, further potentiating the harmful effects of GLAP-RAGE axis in HUVECs. We have previously reported that Gly-AGEs up-regulate RAGE mRNA levels in ECs via RAGE-mediated ROS generation [15,18-20,24]. Guglielmotto *et al*. recently found that GLAP induced RAGE gene expression, and activated a redox-sensitive transcriptional factor, nuclear factor-κB (NF-κB) pathway in neuroblastoma cells, which was prevented by silencing RAGE using RNA interference [35]. Given that RAGE is a major receptor for AGEs that mediates the downstream signaling in ECs and that ROS generation works as a second messenger of RAGE [15,18-20,24], our present study suggests that GLAP could mimic the deleterious effects of Gly-AGEs on HUVECs via the interaction with RAGE, and the positive feedback loop between GLAP-induced ROS generation and RAGE overexpression might make a vicious cycle, promoting vascular damage in diabetes.

In the present study, we also found for the first time that GLAP significantly increased mRNA levels of MCP-1, ICAM-1, VCAM-1 and PAI-1 in HUVECs, which was completely prevented by the treatment with RAGE-Ab. Gly-AGEs are reported to up-regulate MCP-1, ICAM-1, VCAM-1 and PAI-1 gene expression in ECs via ROS generation and transcriptional activation of NF-κB through the interaction with RAGE [16-19,36-38]. Therefore, the GLAP-RAGE axis could evoke inflammatory and thrombogenic reactions in HUVECs by inducing ROS generation. MCP-1 plays an important role in the early phase of atherosclerosis by initiating monocyte recruitment to the vessel wall, and its expression is elevated in human atherosclerotic plaques [39,40]. The selective targeting of CCR2, the receptor for MCP-1, markedly decreases atheromatous lesion formation in apoE knockout mice [39,40]. One early phase of atherosclerosis involves the firm adhesion of inflammatory cells to ECs as well, whose process is mainly mediated by ICAM-1 and VCAM-1 [41-43]. Moreover, attenuated fibrinolytic activity due to increased PAI-1 levels is highly prevalent in diabetic patients, thus contributing to the increased risk of atherothrombosis and cardiovascular disease in these subjects [44,45]. We have previously shown that Gly-AGEs, RAGE and oxidative stress marker, 8-hydroxydeoxyguanosine levels are increased in thoracic aorta of obese and type 2 diabetic rats compared with non-diabetic control rats, all of which are associated with enhanced gene expression of MCP-1, VCAM-1 and PAI-1 and NF-κB activation [46]. Serum levels of Gly-AGEs are an independent determinant of PAI-1 in a general population [47]. Taken together, these observations suggest that activation of the GLAP-RAGE axis could play a role in the progression of atherosclerosis partly via induction of inflammatory and thrombogenic reactions in ECs. The fact that GLAP levels in plasma or tissue are extremely increased in diabetic animals or patients [34,35] further supports the pathological relevance of the GLAP-RAGE axis in accelerated atherosclerosis in diabetes.

### GLAP is a cytotoxic AGE structure in Gly-AGEs

As mentioned above, Gly-AGEs evoked oxidative, inflammatory and thrombogenic reactions in ECs via the interaction with RAGE [15-20]. Furthermore, we have found that Gly-AGEs exist in human serum, and the levels are correlated with endothelial dysfunction and vascular inflammation, surrogate markers for atherosclerotic cardiovascular disease [12,13]. In addition, Gly-AGEs are immunologically distinct from other sugar-derived AGEs or structurally identified AGEs such as glucose-, glycolaldehyde-, fructose-, methylglyoxal-, glyoxal-modified AGEs, carboxymethyllysine-BSA, carboxyethyllysine-BSA, pyrraline-BSA, pentosidine-BSA, argpyrimidine-BSA, and 3-deoxyglucosone imidazolone-BSA [12,48]. These observations suggest that glyceraldehyde-related specific AGE structure in Gly-AGEs might play a role in vascular injury both *in vitro* and *in vivo*. Since GLAP mimicked the deleterious effects of Gly-AGEs on HUVECs, it is conceivable that GLAP is a cytotoxic AGE structure in Gly-AGEs. However, it remains unclear whether AGE-aptamer, which bound to Gly-AGEs, blocked the binding of Gly-AGEs to RAGE and resultantly inhibited the Gly-AGE-evoked oxidative and inflammatory reactions in cell culture and animal models [24-27,30,49], could also inhibit the harmful effects of GLAP on HUVECs. To address the issue, we investigated the effects of AGE-aptamer on GLAP-evoked oxidative stress generation, RAGE, MCP-1, ICAM-1, VCAM-1 and PAI-1 gene induction in HUVECs. In this study, we found that AGE- or GLAP-aptamer, but not Control-aptamer, bound to GLAP and subsequently prevented the interaction of GLAP with RAGE. Moreover, GLAP-evoked increase in ROS generation and up-regulation in RAGE, MCP-1, ICAM-1, VCAM-1 and PAI-1 mRNA levels were significantly blocked by the treatment with AGE- or GLAP-aptamer, but not Control-aptamer. The present findings suggest that GLAP is a target compound for AGE-aptamer and might be a main glyceraldehyde-related AGE structure in Gly-AGEs that interacted with RAGE and subsequently elicited oxidative, inflammatory and thrombogenic reactions in HUVECs. GLAP was only formed in glyceraldehyde-modified BSA, not in other sugar-modified one [34], further supporting our speculation.

Plasma or tissue concentration levels of GLAP in diabetic animals or patients are about 3–4 μg/ml [34,35]. So, the concentration of GLAP (1–10 μg/ml) used here are comparable with those of the *in vivo*-diabetic situation. We did not know how GLAP formation was accelerated under diabetic conditions. However, we, along with others, have previously shown that hyperglycemia-induced overproduction of superoxide by the mitochondrial electron transport chain activates the three major pathways, including increased AGE formation, and causes EC damage by inhibiting glyceraldehyde-3-phosphate dehydrogenase (GAPDH) activity [50,51]. Therefore, although glyceraldehyde, which could be derived from glucose metabolism, is not a major sugar *in vivo*, glyceraldehyde and glyceraldehyde-3 phosphate could be increased under hyperglycemic and oxidative stress conditions via reduced activity of GAPDH, which might lead to promote the formation and accumulation of GLAP in diabetes.

### Limitations

Our study has a couple of limitations that should be noted. First, we did not know whether AGE- or GLAP-aptamer could bind to glucose-derived AGEs and inhibit their biological effects on HUVECs. So, it would interesting to compare the specificity of AGE- or GLAP-aptamer against Gly-AGEs and glucose-derived AGEs, which might confirm the value and specificity of the aptamers. Seconds, we did not examine here the effect of GLAP on atherosclerosis in diabetic animals. Third, although GLAP levels in plasma and tail collagen or brain were reported to increase in diabetic animal [33,34], it remains unknown whether circulating GLAP levels could be a biomarker of vascular injury in diabetic patients. Lastly, AGEs induces cardiomyocyte autophagy by, at least in part, inhibiting the phosphatidylinositol 3-phosphate kinase (PI3K)/Akt/mammalian target of rapamycin (mTOR) pathway via RAGE [52], and AGEs-induced macrophage migration is dependent on heparanase involving RAGE-heparanase-PI3K/AKT pathway [53]. In addition, RAGE activation leads to an increase of transforming growth factor-beta1 levels [54]. Therefore, complex and variable RAGE pathways might be involved in the harmful actions of Gly-AGEs on HUVECs. It would be helpful to examine the effects of GLAP on these signaling pathways in HUVECs. Additional experiments and further clinical studies are needed to clarify the clinical relevance of GLAP-RAGE axis in accelerated atherosclerosis in diabetes.

## Conclusions

Our present observations suggest GLAP might be a main glyceraldehyde-related AGE structure in Gly-AGEs that bound to RAGE and subsequently evoked ROS generation and inflammatory and thrombogenic reactions in HUVECs. Blockade of the GLAP-RAGE interaction by AGE-aptamer or GLAP-aptamer might be a novel therapeutic strategy for preventing vascular injury in diabetes.

## Abbreviations

AGEs: Advanced glycation end products
RAGE: Receptor for AGEs
Gly-AGEs: Glyceraldehyde-derived advanced glycation end products
ECs: Endothelial cells
SELEX: Systematic evolution of ligands by exponential enrichment
AGE-aptamer: DNA aptamer directed against Gly-AGEs
ROS: Reactive oxygen species
HUVECs: Human umbilical vein ECs
GLAP: Glyceraldehyde-derived pyridinium
GLAP-aptamer: DNA aptamer directed against GLAP
BSA: Bovine serum albumin
HPLC: High-performance liquid chromatography
RAGE-Ab: Antibody directed against human RAGE
PCR: Polymerase chain reaction
PBS: Phosphate-buffered saline
Control-aptamer: Control DNA aptamer
DMSO: Dimethyl sulfoxide
RT-PCR: Reverse transcription-PCR
MCP-1: Monocyte chemoattractant protein-1
VCAM-1: Vascular cell adhesion molecule-1
ICAM-1: Intercellular adhesion molecule-1
PAI-1: Plasminogen activator inhibitor-1
vRAGE: extracellular AGE-binding v-domain of RAGE
QCM: Quartz crystal microbalance
*K*_D_: Dissociation constant
NF-κB: Nuclear factor-κB
GAPDH: Glyceraldehyde-3-phosphate dehydrogenase
PI3K: Phosphatidylinositol 3-phosphate kinase
mTOR: mammalian target of rapamycin.

## References

[1] Vlassara H, Bucala R (1996). Recent progress in advanced glycation and diabetic vascular disease: role of advanced glycation end product receptors. Diabetes.

[2] Brownlee M, Cerami A, Vlassara H (1988). Advanced glycosylation end products in tissue and the biochemical basis of diabetic complications. N Engl J Med.

[3] Rahbar S (2007). Novel inhibitors of glycation and AGE formation. Cell Biochem Biophys.

[4] Yamamoto Y, Kato I, Doi T, Yonekura H, Ohashi S, Takeuchi M, Watanabe T, Yamagishi S, Sakurai S, Takasawa S, Okamoto H, Yamamoto H (2001). Development and prevention of advanced diabetic nephropathy in RAGE-overexpressing mice. J Clin Invest.

[5] Wendt TM, Tanji N, Guo J, Kislinger TR, Qu W, Lu Y, Bucciarelli LG, Rong LL, Moser B, Markowitz GS, Stein G, Bierhaus A, Liliensiek B, Arnold B, Nawroth PP, Stern DM, D'Agati VD, Schmidt AM (2003). RAGE drives the development of glomerulosclerosis and implicates podocyte activation in the pathogenesis of diabetic nephropathy. Am J Pathol.

[6] Yamagishi S, Imaizumi T (2005). Diabetic vascular complications: pathophysiology, biochemical basis and potential therapeutic strategy. Curr Pharm Des.

[7] Yamagishi S, Nakamura K, Matsui T, Noda Y, Imaizumi T (2008). Receptor for advanced glycation end products (RAGE): a novel therapeutic target for diabetic vascular complication. Curr Pharm Des.

[8] Jandeleit-Dahm K, Cooper ME (2008). The role of AGEs in cardiovascular disease. Curr Pharm Des.

[9] Jinnouchi Y, Yamagishi S, Takeuchi M, Ishida S, Jinnouchi Y, Jinnouchi J, Imaizumi T (2006). Atorvastatin decreases serum levels of advanced glycation end products (AGEs) in patients with type 2 diabetes. Clin Exp Med.

[10] Hyogo H, Yamagishi S, Iwamoto K, Arihiro K, Takeuchi M, Sato T, Ochi H, Nonaka M, Nabeshima Y, Inoue M, Iahitobi T, Chayama K, Tazuma S (2007). Elevated levels of serum advanced glycation end products in patients with non-alcoholic steatohepatitis. J Gastroenterol Hepatol.

[11] Tahara N, Yamagishi S, Matsui T, Takeuchi M, Nitta Y, Kodama N, Mizoguchi M, Imaizumi T (2012). Serum levels of advanced glycation end products (AGEs) are independent correlates of insulin resistance in nondiabetic subjects. Cardiovasc Ther.

[12] Tahara N, Yamagishi S, Takeuchi M, Honda A, Tahara A, Nitta Y, Kodama N, Mizoguchi M, Kaida H, Ishibashi M, Hayabuchi N, Matsui T, Imaizumi T (2012). Positive association between serum level of glyceraldehyde-derived advanced glycation end products and vascular inflammation evaluated by [(18)F]fluorodeoxyglucose positron emission tomography. Diabetes Care.

[13] Kajikawa M, Nakashima A, Fujimur N, Maruhashi T, Iwamoto Y, Iwamoto A, Matsumoto T, Oda N, Hidaka T, Kihara Y, Chayama K, Goto C, Aibara Y, Noma K, Takeuchi M, Matsui T, Yamagishi S, Higashi Y (2015). Ratio of serum levels of AGEs to soluble form of RAGE is a predictor of endothelial function. Diabetes Care.

[14] Ueda S, Yamagishi S, Matsui T, Noda Y, Ueda S, Jinnouchi Y, Sasaki K, Takeuchi M, Imaizumi T (2012). Serum levels of advanced glycation end products (AGEs) are inversely associated with the number and migratory activity of circulating endothelial progenitor cells in apparently healthy subjects. Cardiovasc Ther.

[15] Yamagishi S, Nakamura K, Matsui T, Inagaki Y, Takenaka K, Jinnouchi Y, Yoshida Y, Matsuura T, Narama I, Motomiya Y, Takeuchi M, Inoue H, Yoshimura A, Bucala R, Imaizumi T (2006). Pigment epithelium-derived factor inhibits advanced glycation end product-induced retinal vascular hyperpermeability by blocking reactive oxygen species-mediated vascular endothelial growth factor expression. J Biol Chem.

[16] Yamagishi S, Matsui T, Nakamura K, Inoue H, Takeuchi M, Ueda S, Okuda S, Imaizumi T (2008). Olmesartan blocks inflammatory reactions in endothelial cells evoked by advanced glycation end products by suppressing generation of reactive oxygen species. Ophthalmic Res.

[17] Ishibashi Y, Matsui T, Takeuchi M, Yamagishi S (2010). Glucagon-like peptide-1 (GLP-1) inhibits advanced glycation end product (AGE)-induced up-regulation of VCAM-1 mRNA levels in endothelial cells by suppressing AGE receptor (RAGE) expression. Biochem Biophys Res Commun.

[18] Ishibashi Y, Matsui T, Maeda S, Higashimoto Y, Yamagishi S (2013). Advanced glycation end products evoke endothelial cell damage by stimulating soluble dipeptidyl peptidase-4 production and its interaction with mannose 6-phosphate/insulin-like growth factor II receptor. Cardiovasc Diabetol.

[19] Ishibashi Y, Matsui T, Ueda S, Fukami K, Yamagishi S (2014). Advanced glycation end products potentiate citrated plasma-evoked oxidative and inflammatory reactions in endothelial cells by up-regulating protease-activated receptor-1 expression. Cardiovasc Diabetol.

[20] Ishibashi Y, Matsui T, Takeuchi M, Yamagishi S (2011). Sitagliptin augments protective effects of GLP-1 against advanced glycation end product receptor axis in endothelial cells. Horm Metab Res.

[21] Ellington AD, Szostak JW (1990). In vitro selection of RNA molecules that bind specific ligands. Nature.

[22] Gragoudas ES, Adamis AP, Cunningham ET, Feinsod M, Guyer DR (2004). Group VISiONCT: Pegaptanib for neovascular age-related macular degeneration. N Engl J Med.

[23] Jilma-Stohlawetz P, Gilbert JC, Gorczyca ME, Knöbl P, Jilma B (2011). A dose ranging phase I/II trial of the von Willebrand factor inhibiting aptamer ARC1779 in patients with congenital thrombotic thrombocytopenic purpura. Thromb Haemost.

[24] Higashimoto Y, Matsui T, Nishino Y, Taira J, Inoue H, Takeuchi M, Yamagishi S (2013). Blockade by phosphorothioate aptamers of advanced glycation end products-induced damage in cultured pericytes and endothelial cells. Microvasc Res.

[25] Kaida Y, Fukami K, Matsui T, Higashimoto Y, Nishino Y, Obara N, Nakayama Y, Ando R, Toyonaga M, Ueda S, Takeuchi M, Inoue H, Okuda S, Yamagishi S (2013). DNA aptamer raised against AGEs blocks the progression of experimental diabetic nephropathy. Diabetes.

[26] Ojima A, Oda E, Higashimoto Y, Matsui T, Yamagishi S (2014). DNA aptamer raised against advanced glycation end products inhibits neointimal hyperplasia in balloon-injured rat carotid arteries. Int J Cardiol.

[27] Ojima A, Matsui T, Maeda S, Takeuchi M, Inoue H, Higashimoto Y, Yamagishi S (2014). DNA aptamer raised against advanced glycation end products inhibits melanoma growth in nude mice. Lab Invest.

[28] Usui T, Hayase F (2003). Isolation and identification of the 3-hydroxy-5-hydroxymethyl-pyridinium compound as a novel advanced glycation end product on glyceraldehyde-related Maillard reaction. Biosci Biotechnol Biochem.

[29] Sasaki N, Takeuchi M, Chowei H, Kikuchi S, Hayashi Y, Nakano N, Ikeda H, Yamagishi S, Kitamoto T, Saito T, Makita Z (2002). Advanced glycation end products (AGE) and their receptor (RAGE) in the brain of patients with Creutzfeldt-Jakob disease with prion plaques. Neurosci Lett.

[30] Higashimoto Y, Yamagishi S, Nakamura K, Matsui T, Takeuchi M, Noguchi M, Inoue H (2007). In vitro selection of DNA aptamers that block toxic effects of AGE on cultured retinal pericytes. Microvasc Res.

[31] Nakashima S, Matsui T, Yamagishi S (2013). Pigment epithelium-derived factor (PEDF) blocks high glucose-induced inflammatory reactions in endothelial cells through its anti-oxidative properties. Int J Cardiol.

[32] Zuker M (2013). Mfold web server for nucleic acid folding and hybridization prediction. Nucleic Acids Res.

[33] Efremov V, Killard AJ, Byrne B, Lakshmanan RS (2013). The modelling of blood coagulation using the quartz crystal microbalance. J Biomech.

[34] Usui T, Shimohira K, Watanabe H, Hayase F (2007). Detection and determination of glyceraldehyde-derived pyridinium-type advanced glycation end products in streptozotocin-induced diabetic rats. Biosci Biotechnol Biochem.

[35] Guglielmotto M, Aragno M, Tamagno E, Vercellinatto I, Visentin S, Medana C, Catalano MG, Smith MA, Perry G, Danni O, Boccuzzi G, Tabaton G (2012). AGEs/RAGE complex upregulates BACE1 via NF-κB pathway activation. Neurobiol Aging.

[36] Tanaka N, Yonekura H, Yamagishi S, Fujimori H, Yamamoto Y, Yamamoto H (2000). The receptor for advanced glycation end products is induced by the glycation products themselves and tumor necrosis factor-alpha through nuclear factor-kappa B, and by 17beta-estradiol through Sp-1 in human vascular endothelial cells. J Biol Chem.

[37] Okamoto T, Yamagishi S, Inagaki Y, Amano S, Koga K, Abe R, Takeuchi M, Ohno S, Yoshimura A, Makita Z (2002). Angiogenesis induced by advanced glycation end products and its prevention by cerivastatin. FASEB J.

[38] Yamagishi S, Matsui T, Nakamura K, Yoshida T, Takeuchi M, Inoue H, Yoshida Y, Imaizumi T (2007). Pigment-epithelium-derived factor suppresses expression of receptor for advanced glycation end products in the eye of diabetic rats. Ophthalmic Res.

[39] Gu L, Okada Y, Clinton SK, Gerard C, Sukhova GK, Libby P, Rollins BJ (1998). Absence of monocyte chemoattractant protein-1 reduces atherosclerosis in low density lipoprotein receptor-deficient mice. Mol Cell.

[40] Boring L, Gosling J, Cleary M, Charo IF (1998). Decreased lesion formation in CCR2−/− mice reveals a role for chemokines in the initiation of atherosclerosis. Nature.

[41] Lawson C, Wolf S (2009). ICAM-1 signaling in endothelial cells. Pharmacol Rep.

[42] Preiss DJ, Sattar N (2007). Vascular cell adhesion molecule-1: a viable therapeutic target for atherosclerosis?. Int J Clin Pract.

[43] Matsui T, Higashimoto Y, Taira J, Yamagishi S (2013). Pigment epithelium-derived factor (PEDF) binds to caveolin-1 and inhibits the pro-inflammatory effects of caveolin-1 in endothelial cells. Biochem Biophys Res Commun.

[44] Takenaka K, Yamagishi S, Matsui T, Nakamura K, Imaizumi T (2006). Role of advanced glycation end products (AGEs) in thrombogenic abnormalities in diabetes. Curr Neurovasc Res.

[45] Yamagishi S, Fujimori H, Yonekura H, Yamamoto Y, Yamamoto H (1998). Advanced glycation endproducts inhibit prostacyclin production and induce plasminogen activator inhibitor-1 in human microvascular endothelial cells. Diabetologia.

[46] Matsui T, Nishino Y, Takeuchi M, Yamagishi S (2011). Vildagliptin blocks vascular injury in thoracic aorta of diabetic rats by suppressing advanced glycation end product-receptor axis. Pharmacol Res.

[47] Yamagishi S, Adachi H, Takeuchi M, Enomoto M, Furuki K, Matsui T, Nakamura K, Imaizumi T (2007). Serum level of advanced glycation end-products (AGEs) is an independent determinant of plasminogen activator inhibitor-1 (PAI-1) in nondiabetic general population. Horm Metab Res.

[48] Takeuchi M, Makita Z, Bucala R, Suzuki T, Koike T, Kameda Y (2000). Immunological evidence that non-carboxymethyllysine advanced glycation end-products are produced from short chain sugars and dicarbonyl compounds in vivo. Mol Med.

[49] Ojima A, Matsui T, Nakamura N, Higashimoto Y, Ueda S, Fukami K, Okuda S, Yamagishi S: **DNA Aptamer Raised Against Advanced Glycation End Products (AGEs) Improves Glycemic Control and Decreases Adipocyte Size in Fructose-Fed Rats by Suppressing AGE-RAGE Axis**. *Horm Metab Res* 2014, doi:10.1055/s-0034-1385904 10.1055/s-0034-1385904 25105541

[50] Nishikawa T, Edelstein D, Du XL, Yamagishi S, Matsumura T, Kaneda Y, Yorek MA, Beebe D, Oates PJ, Hammes HP, Giardino I, Brownlee M (2000). Normalizing mitochondrial superoxide production blocks three pathways of hyperglycaemic damage. Nature.

[51] Du X, Matsumura T, Edelstein D, Rossetti L, Zsengellér Z, Szabó C, Brownlee M (2003). Inhibition of GAPDH activity by poly(ADP-ribose) polymerase activates three major pathways of hyperglycemic damage in endothelial cells. J Clin Invest.

[52] Hou X, Hu Z, Xu H, Xu J, Zhang S, Zhong Y, He X, Wang N (2014). Advanced glycation endproducts trigger autophagy in cadiomyocyte via RAGE/PI3K/AKT/mTOR pathway. Cardiovasc Diabetol.

[53] Qin Q, Niu J, Wang Z, Xu W, Qiao Z, Gu Y (2013). Heparanase induced by advanced glycation end products (AGEs) promotes macrophage migration involving RAGE and PI3K/AKT pathway. Cardiovasc Diabetol.

[54] Serban AI, Stanca L, Geicu OI, Munteanu MC, Costache M, Dinischiotu A: **Extracellular matrix is modulated in advanced glycation end products milieu via a RAGE receptor dependent pathway boosted by transforming growth factor-β1.** *J Diabetes* 2014, doi:10.1111/1753-0407 10.1111/1753-0407.12154 24666836

